# *TRAF1/C5 *polymorphism is not associated with increased mortality in rheumatoid arthritis: two large longitudinal studies

**DOI:** 10.1186/ar2947

**Published:** 2010-03-05

**Authors:** Jessica AB van Nies, Rute B Marques, Stella Trompet, Zuzana de Jong, Fina AS Kurreeman, Rene EM Toes, J Wouter Jukema, Tom WJ Huizinga, Annette HM van der Helm-van Mil

**Affiliations:** 1 Department of Rheumatology, Leiden University Medical Centre, Albinusdreef 2, 2333 ZA Leiden, The Netherlands; 2 Department of Cardiology, Leiden University Medical Centre, Albinusdreef 2, 2333 ZA Leiden, The Netherlands; 3 Department of Gerontology and Geriatrics, Leiden University Medical Centre, Albinusdreef 2, 2333 ZA Leiden, The Netherlands

## Abstract

**Introduction:**

Recently an association between a genetic variation in *TRAF1/C5 *and mortality from sepsis or cancer was found in rheumatoid arthritis (RA). The most prevalent cause of death, cardiovascular disease, may have been missed in that study, since patients were enrolled at an advanced disease stage. Therefore, we used an inception cohort of RA patients to investigate the association between *TRAF1/C5 *and cardiovascular mortality, and replicate the findings on all-cause mortality. As *TRAF1/C5 *associated mortality may not be restricted to RA, we also studied a large cohort of non-RA patients.

**Methods:**

615 RA patients from the Leiden Early Arthritis Clinic (EAC) (mean follow-up 7.6 years) were genotyped for rs10818488. In addition 5634 persons enrolled in the PROspective Study of Pravastatin in the Elderly at Risk (mean follow-up 3.2 years) were genotyped for rs2416808 (R^2 ^>0.99 with rs10818488). The life/death status was determined and for the deceased persons the cause of death was ascertained. Cox proportional hazards and regression models were used to assess hazard ratios (HR) and 95% confidence intervals (CI).

**Results:**

Seventy-seven RA patients died. The main death causes in RA patients were cardiovascular diseases (37.7%), cancer (28.6%) and death due to infections (9.1%). No association was observed between the rs10818488 susceptible genotype AA and cardiovascular mortality (HR 1.08 95%CI 0.54 to 2.15) and all-cause mortality (HR 0.81 95%CI 0.27 to 2.43). Similar findings were observed for rs2416808 susceptible genotype GG in the non-RA cohort (HR 0.99; 95%CI 0.79 to 1.25 and HR 0.89; 95%CI 0.64 to 1.25, respectively).

**Conclusions:**

The *TRAF1/C5 *region is not associated with an increased mortality risk.

## Introduction

Patients with rheumatoid arthritis (RA) have an increased mortality risk. A recent review, studying data from 84 unique cohorts, showed that mortality rates in RA patients were 1.5 to 1.6 fold higher than in the general population [1]. The attributed causes of death in RA patients are identical to those in the general population [1], cardiovascular disease being the primary cause of death followed by cancer and infection. Age, sex and most clinical markers that are related to a more severe destructive disease course (among others number of inflamed joints, C reactive protein (CRP) and presence of erosions) are also associated with higher mortality risks [1].

Genetic risk factors for mortality in RA are scarcely investigated. Presence of the human leukocyte antigen (HLA)-DRB1 shared epitope alleles are reported to be associated with an increased mortality and, in particular, mortality related to cardiovascular disease (CVD) [2-4]. Although a lot of progress has been made in the field of genetics of RA-susceptibility, the HLA-shared epitope alleles still constitute the most powerful genetic risk factor to developing RA. Well-replicated non-HLA risk factors are *PTPN22, TNFAIP3*, and *TRAF1/C5 *[5-7]. *TRAF1/C5 *associated with several autoimmune diseases other than RA such as juvenile idiopathic arthritis (JIA) and systemic lupus erythomatodes [8,9].

A recent study analyzed the *TRAF1/C5 *variant, rs3761847, in relation to mortality in RA and observed an increased mortality risk for the susceptible genotype GG compared with the non-susceptible AA genotype (hazard ratio (HR) = 3.96, 95% confidence interval (CI) = 1.24 to 12.6) [10]. Such an observation is relevant because it indicates that genetic factors, such as *TRAF1/C5*, contribute to the increased mortality in RA. The causes of death were also investigated. Intriguingly, no increased death rate for CVD was found among GG homozygotes, whereas increased death rates were found for cancer and infections. As this study enrolled patients with a median disease duration of 10 (range 4 to 18) years, which were subsequently followed up for two to four years, CVD-related deaths occurring earlier in the disease course may have been missed. To further explore the association between the *TRAF1/C5 *locus and CVD-related mortality in RA, we studied a cohort of 615 early RA patients that were followed from disease onset to 14 years onwards. In addition, we investigated whether the association with all-cause, infectious and cancer-related mortality Panoulas and colleagues observed could be replicated [10]. Finally, as an association between *TRAF1/C5 *and mortality may not be restricted to RA patients, we also analysed a large cohort of non-RA patients.

## Materials and methods

### Early arthritis clinic cohort

The Leiden early arthritis cohort (EAC) is an inception cohort consisting of patients with recent-onset arthritis referred to the Department of Rheumatology of the Leiden University Medical Center from 1993 onwards [11]. Patients were included when arthritis was observed by a rheumatologist. For the present study, patients were selected who fulfilled the American College of Rheumatology 1987 revised criteria for RA within the first year of follow up and had DNA samples available (n = 615) [12]. Written informed consent was obtained from all participants. The study was approved by the appropriate local institutional review board. At inclusion, a physical examination was performed and blood samples were taken to determine CRP, immunoglobulin (Ig) M rheumatoid factor (RF; by ELISA) and anti-cyclic citrullinated peptide (CCP) 2 antibodies (Immunoscan RA Mark 2; Euro-Diagnostica, Arnhem, the Netherlands). Anti-CCP2 positivity had a cut-off level of 25 arbitrary units, according to manufacturer's instructions. Patients also filled in a Health Assessment Questionnaire (HAQ) [13] and radiographs of hands and feet were taken and scored by one experienced reader (the intraclass-observer correlation coefficients was 0.91), using the Sharp-van der Heijde method [14].

### Cohort of non-RA patients

As well as the RA patients, 5634 participants with available DNA from the PROspective Study of Pravastatin in the Elderly at Risk (PROSPER) were studied. In short, PROSPER is a randomized double-blind placebo-controlled trial that assessed whether pravastatin treatment in elderly men and women diminished the risk of major vascular events [15]. Participants were screened and enrolled in Scotland (Glasgow), Ireland (Cork), and the Netherlands (Leiden) between December 1997 and May 1999. Written informed consent was obtained from all participants. The study was approved by the appropriate local institutional review boards of all centers. The primary endpoint in the study was a combination of death from coronary heart disease (CHD), non-fatal myocardial infarction (MI), and fatal or non-fatal stroke. After three years of follow up, CVD and transient ischemic attacks (TIAs) were less prevalent in the group treated with pravastatin [16].

### SNP genotyping

rs10818488 was genotyped in the RA cohort and rs2416808, which is in complete linkage disequilibrium with rs10818488 (r^2^>0.99), was genotyped in the non-RA cohort. Genotypings were performed using the MassArray matrix-assisted laser desorption ionisation time-of-flight mass spectrometry, according to the protocols recommended by the manufacturer (Sequenom, San Diego, CA, USA). Each 384-well plate contained at least 4 positive (CEPH DNA) and 4 negative controls, to check for assay performance and contaminations, respectively. SpectroCaller software (Sequenom, San Diego, CA, USA) supplied by the manufacturer was used to automatically identify the genotypes. Clusters were checked and all doubtful calls were manually evaluated. Ten percent of the genotypes were performed in duplicate and the error rate was below 1%. Allele frequencies and Hardy-Weinberg equilibrium consistency were determined with Haploview [17]. Both SNPs were in agreement with Hardy-Weinberg equilibrium. Both rs10818488 and rs2416808 are in complete linkage-disequilibrium with rs3761847 genotyped by Panoulas and colleagues (r^2^>0.99 data from Hapmap, and Kurreeman and colleagues) [5,6,10].

### Notification of death

In the EAC, patients were followed longitudinally from the moment of their inclusion until 1 April, 2008, or death. All RA patients were tracked nationally using the civic registries (Gemeentelijke Basis Administratie) to ascertain life or death status. Causes of death for RA patients were obtained from Statistics Netherlands [18] and coded according to the International Classification of Diseases 10^th ^revision of the World Health Organization [19]. In the non-RA cohort, patients were followed for 3.2 years and the causes of death in this period were obtained from post-mortem reports and/or certification of death. All endpoints were adjudicated by a study endpoint committee.

### Analysis of data

Data are expressed as mean (± standard deviation (SD)) with a 95% CI for continuous variables and as proportions for categorical variables. Differences in baseline patient characteristics between the single nucleotide protocol (SNP) genotypes were compared using a one-way analysis of variance test or Kruskal Wallis test for continuous variables and the chi-squared test for nominal variables. Associations between genotype and mortality were tested with univariate cox regression analyses and log rank tests.

In a study in RA patients (unpublished) we observed that age, CRP level and Sharp-van der Heijde score were independently associated with mortality. In order to investigate whether *TRAF1/C5 *is associated with mortality after adjusting for gender, HAQ-score and other previously found risk factors, a multivariate cox regression analysis was performed.

Subjects from the cohort of non-RA patients who withdrew consent or died during the study were censored at the date of death or at the last date of follow up. Cox regression analyses in the cohort of non-RA patients were adjusted for gender, age, pravastatin or placebo use, and country.

Panoulas and colleagues observed a HR of 3.96. The RA cohort has a power of 99.6% to identify such an association with *C5/TRAF1 *based on the observed genotype frequencies and an alpha of 0.05. In the non-RA cohort this power was 100%. Assuming that the finding by Panoulas and colleagues was affected by the winners curse and the true HR would be lower, for example a HR of 1.5, then the power of the RA cohort and non-RA cohort to observe an association was 28% and 97.5%, respectively.

All statistical analyses were performed using Statistical Package for Social Sciences version 16.0 (SPSS, Chicago, IL, USA). In all tests, *P *values below 0.05 were considered to be significant.

## Results

### Baseline characteristics

The 615 RA patients had mean (± SD) age of 56.4 ± 15.6 years and 68.5% were female. The mean CRP concentration was 29.5 ± 33.1 mg/L, 57% were RF positive, 43.4% were anti-CCP2 positive, the mean HAQ score was 1.07 ± 0.72 and the median Sharp-van der Heijde score at baseline was 6 (range 2 to 12). The genotype frequencies for rs10818488 were: 29% GG (n = 180), 53% AG (n = 324) and 18% AA (n = 111). No differences in baseline characteristics between genotypes were observed (Table 1). The mean follow up was 7.6 ± 3.6 years (range 4.7 to 10.5 years).

**Table 1:** Baseline characteristics of the RA patients per genotype of rs10818488

	GG n = 180 (29%)	AG n = 324 (53%)	AA n = 111 (18%)
Age (years)	56.2 ± 15.2	56.3 ± 15.5	57.3 ± 16.5
Females, n (%)	132 (73.3)	218 (67.3)	71 (64.0)
Past or current smoker, n (%)	83 (51.2)	139 (48.1)	53 (54.1)
Swollen joint count	9.0 ± 6.6	9.4 ± 7.3	8.0 ± 6.0
BMI (kg/m^2^)	25.5 ± 3.7	25.7 ± 3.8	25.7 ± 4.0
RF positive, n (%)	101 (57.1)	181 (57.3)	70 (63.6)
Anti-CCP positive, n (%)	75 (56.4)	146 (57.5)	46 (51.7)
CRP (mg/L)	28.1 ± 29.0	31.6 ± 36.1	25.8 ± 30.1
HAQ (0-3)	1.05 ± 0.70	1.10 ± 0.72	1.03 ± 0.77
Total Sharp- van der Heijde score, median (IQR 25-75)	6 (2-13)	6 (2-12)	5 (1.5-11)

Except if stated otherwise values are mean (standard deviation).

#Data not available for all cases (smoking status n = 66, swollen joint count n = 111, BMI n = 156, rheumatoid factor in n = 12, anti CCP n = 139, CRP n = 46, HAQ n = 113, Total Sharp- van der Heijde score n = 27).

BMI, body mass index; CCP, cyclic citrullinated peptide; CRP, C-reactive protein; IQR, interquartile range; HAQ, Health Assessment Questionnaire; RA, rheumatoid arthritis; RF, rheumatoid factor.

In the cohort of non-RA patients 51.6% were female and the mean age was 75.3 ± 3.4 years. For baseline characteristics see Table 2. The slight difference in history of stroke was considered to be a spurious finding due to multiple comparisons. The genotype frequencies for rs2416808 were: 30.9% AA (n = 1743), 48.4% AG (n = 2725) and 19.9% GG (n = 1123). The mean follow up in these patients was 3.2 ± 0.6 years.

**Table 2:** Baseline characteristics of the non-RA patients per genotype of rs2416808

	AA n = 1783 (32%)	AG n = 2725 (48%)	GG n = 1123 (20%)
Age (years)	75.24 (3.35)	75.39 (3.31)	75.35 (3.45)
Females, n (%)	898 (50)	1427 (52)	582 (52)
Current smoker, n (%)	459 (26)	738 (27)	311 (28)
BMI (kg/m^2^)	26.85 (4.13)	26.85 (4.19)	26.76 (4.28)
Systolic blood pressure (mmHg)	154.36 (21.18)	154.78 (22.23)	154.89 (21.59)
Diastolic blood pressure (mmHg)	83.65 (11.34)	83.97 (11.45)	83.50 (11.67)
CRP (mg/L)	5.67 (8.65)	5.97 (11.58)	6.13 (13.32)
Total cholesterol (mmol/L)	5.68 (0.90)	5.66 (0.90)	5.74 (0.92)
History of diabetes, n (%)	203 (11)	281 (10)	113 (10)
History of hypertension, n (%)	1093 (61)	1721 (63)	678 (60)
History of myocardial infarction, n (%)	239 (13)	375 (14)	146 (13)
History of stroke or TIA, n (%)	174 (10)	321 (12)	137 (12)*
History of vascular disease, n (%)	770 (43)	1220 (45)	502 (45)
History of angina, n (%)	484 (27)	740 (27)	290 (26)
History of claudication, n (%)	124 (7)	180 (7)	75 (7)

Except if stated otherwise values are mean (standard deviation).

* History of TIA: *P *value 0.027.

BMI, body mass index; CRP, C-reactive protein; RA, rheumatoid arthritis; TIA, transient ischemic attack.

### Mortality in RA cohort

**Figure 1:**
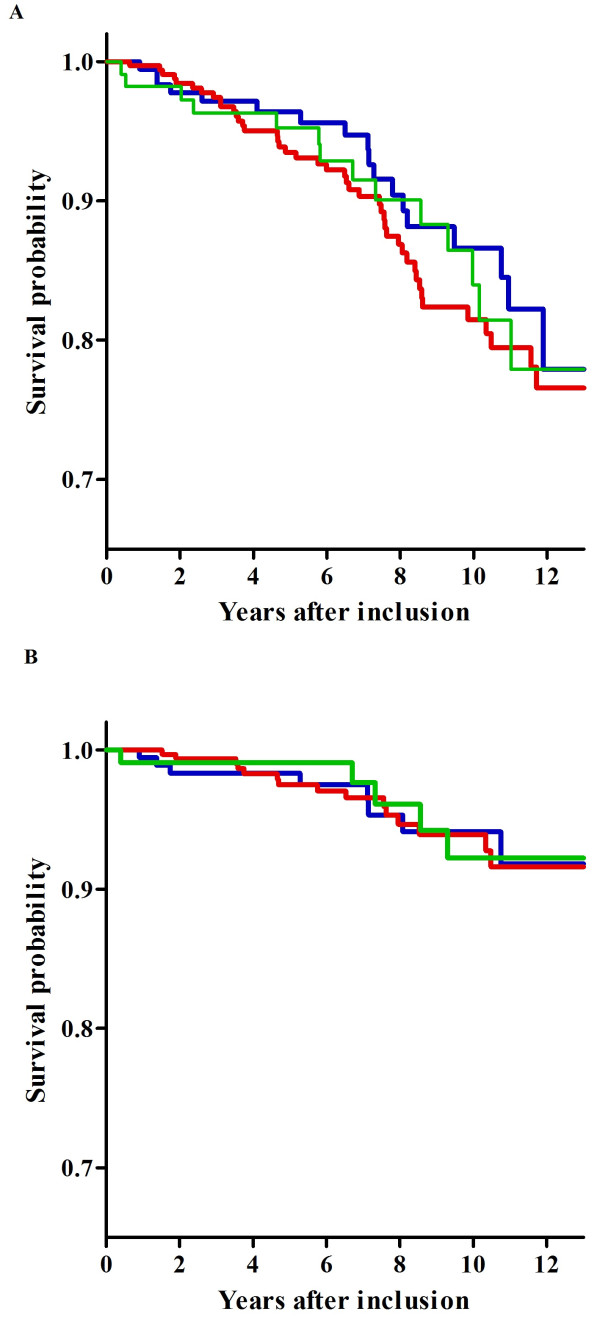
**Survival curves for all-cause and cardiovascular mortality in RA patients per genotype of rs10818488**. The log rank tests showed *P *values for all-cause mortality and cardiovascular mortality of 0.69 and 0.93, respectively. **(a) **All-cause mortality. **(b) **Cardiovascular mortality. Blue line: GG n = 180. Red line: AG n = 324. Green line: AA n = 111.

Seventy-seven RA patients died during follow up; 46 (11%) women and 31 (16%) men. Twenty-five percent of these patients carried the GG genotype, 57% the AG genotype and 18% the AA genotype. The survival probability of rs10818488 genotypes and all-cause mortality is presented in Figure 1a. No significant difference in all-cause mortality was found across the genotypes (HR = 1.06, 95% CI = 0.76 to 1.46, P = 0.752). The major cause of death in RA patients was attributed to CVD (37.7%). This was most frequently caused by acute MI and the second most frequent cause of CVD-related mortality was heart failure. Besides CVD, two other major causes of death in RA patients were cancer (28.6%; n = 22), most frequently of the bronchus and lungs, and infection (9.1%; n = 7).

The 29 patients who died due to CVD had the following genotypes for rs10818488: 31% GG homozygotes, 52% AG heterozygotes and 17% AA homozygotes. Survival probability of CVD-related mortality is presented in Figure 1b. Also, here no significant difference was found between the three genotypes (HR = 0.91, 95% CI = 0.53-1.54, *P *= 0.713; Table 3).

**Table 3:** Hazard ratios for genotypes rs10818488 and rs2416808 in the RA-cohort and Non-RA cohort in univariate cox regression analysis

Hazard ratios RA cohort				
	**Trend**		**AA vs GG**	
		
**Mortality**	**HR(95% CI)**	***P *value**	**HR (95% CI)**	***P *value**

All-cause	1.06 (0.76-1.46)	0.752	1.08 (0.54-2.15)	0.830
CVD	0.91 (0.53-1.54)	0.713	0.81 (0.27-2.43)	0.712
Cancer	0.90 (0.49-1.67)	0.741	0.73 (0.18-2.92)	0.657
Infectious	1.19 (0.41-3.51)	0.748	1.42 (0.09-22.7)	0.803

**Hazards ratios non-RA cohort**				

	**Trend**		**GG vs AA**	
		
**Mortality**	**HR (95% CI)**	***P *value**	**HR (95% CI)**	***P *value**

All-cause	0.99 (0.89-1.11)	0.890	0.99 (0.79-1.25)	0.924
CVD	0.95 (0.80-1.12)	0.512	0.89 (0.64-1.25)	0.515
Cancer	1.00 (0.82-1.21)	0.980	1.02 (0.70-1.50)	0.919

CI, confidence interval; HR, hazard ratio; CVD, cardiovascular disease; RA, rheumatoid arthritis.

Multivariate cox regression analyses were performed to assess whether the *TRAF1/C5 *susceptible genotype was associated with mortality after adjustments for other known risk factors for mortality. Also after adjustments, no significant association between rs10818488 genotype and all-cause (HR = 0.91, 95% CI = 0.61 to 1.37), cardiovascular (HR = 0.97, 95% CI = 0.54 to 1.76), cancer (HR = 0.66, 95% CI = 0.28 to 1.56) or infectious-related mortality (HR = 0.54, 95% CI = 0.09 to 3.13) was observed.

### Mortality in non-RA cohort

In the non-RA cohort, 586 participants died, 32% were AA homozygote, 48% AG heterozygote and 20% GG homozygote for rs2416808. Also, no significant association was found between the *TRAF1/C5 *variants and all-cause mortality (HR = 0.99, 95% CI = 0.89 to 1.11), CVD-related (HR = 0.95, 95% CI = 0.80 to 1.12) and cancer-related mortality (HR = 1.00, 95% CI = 0.82 to 1.21; Table 3). Analyzing only the placebo-treated group did not change the results (data not shown). Similarly, also in the non-RA cohort after adjustments, no significant association was found between rs2416808 and all-cause (HR = 0.99, 95% CI = 0.88 to 1.11), cardiovascular (HR = 0.94, 95% CI = 0.80 to 1.11) or cancer-related mortalities (HR = 1.00, 95% CI = 0.82 to 1.22) in a multivariate cox regression analysis.

## Discussion

The present study was performed to investigate the relation between a well-replicated genetic RA susceptibility factor, *TRAF1/C5*, and the mortality risk in a cohort of RA patients. No evidence for an association of this risk factor with mortality was observed.

Panoulas and colleagues recently observed an increased mortality risk for carriers of the *TRAF1/C5 *susceptibility risk genotype in RA patients [10]. Both polymorphisms analyzed in the present study, rs10818488 and rs2416808, are in complete linkage-disequilibrium with the rs3761847 SNP genotyped by Panoulas and colleagues (r^2^>0.99 data from Hapmap, and Kurreeman and colleagues) [5,6]. However, we could not replicate their finding, despite having a larger cohort (615 vs 400 RA patients), a longer follow-up duration (mean 7.6 vs 2.6 years) and a higher number of events (77 vs 23 deaths). In addition, Panoulas and colleagues reported an increased risk of death due to cancer and sepsis, but not CVD. It is possible that the cohort design from Panoulas and colleagues was less suitable to study association with CVD-related mortality, because patients were enrolled at an advanced disease stage. In this setting, a possible relation between *TRAF1/C5 *and cardiovascular mortality may be missed, because patients dying of CVD earlier in the disease course are not part of their cohort. This hypothesis was corroborated by our results in the RA cohort, which showed that most of the CVD-related deaths were concentrated in the first 10 years of disease. However, also in our EAC, no association was found between *TRAF1/C5 *locus and cardiovascular mortality in RA.

In order to further unravel the eventual association between *TRAF1/C5 *locus and mortality, we hypothesised that this risk would not be restricted to RA. Therefore, we also genotyped *TRAF1/C5 *in a large cohort of elderly people that were prospectively followed in PROSPER. This large cohort was well-powered to detect also small HR (e.g. 1.5). Nonetheless, *TRAF1/C5 *did not confer an increased mortality risk.

This strengthened our findings of an absent association between *TRAF1/C5 *and risk of death.

In the end, one might argue that any cohort that enrolls older participants is inappropriate to study mortality, because the risk genotype may cause an early death. In this sense, prospective studies that follow the participants over a period of decades may be more suitable to answer this question. Nevertheless, the rs10818488 genotype frequencies in our JIA cohort (GG = 30%, AG = 53%, AA = 17%), which has a mean age of 6.4 years at inclusion, did not differ from the frequencies in the adult EAC cohort [8]. Also the minor allele frequency in the non-RA cohort is similar to those in the general population [20]. In addition, both cohorts were in agreement with Hardy-Weinberg equilibrium. This indicates that there was no selection of the protective genotype in the RA or non-RA cohort. This makes an early death in the risk genotype carriers unlikely and further supports the lack of association of these *TRAF1/C5 *variants with mortality.

In the RA-cohort, mortality due to infections were relatively infrequent, the number of deaths attributed to infections is insufficient to make definite conclusions on the association between *TRAF1/C5 *and this specific cause of mortality.

The important causes of death observed in RA patients in the present study are similar to the main causes of death in the general Dutch population [18]. The frequency of CVD-related mortality itself is reported to be higher in RA patients than in healthy individuals [1]. Results from the Nurses Health Study revealed that women with RA had a relative risk of 1.8 for fatal MI [21]. Unfortunately, we were unable to test whether the frequency of cardiovascular death was also increased in our EAC RA cohort, because we do not have mortality information on an age- and gender-matched Dutch control population.

## Conclusions

In conclusion, *TRAF1/C5 *polymorphisms predisposing to RA susceptibility are not associated with all-cause mortality or cardiovascular- or cancer-related mortality in RA and in an elderly cohort of persons without RA.

## Abbreviations

CCP: cyclic citrullinated peptide; CHD: coronary heart disease; CI: confidence interval; CRP: C reactive protein; CVD: cardiovascular disease; EAC: early arthritis clinic; ELISA: enzyme-linked immunosorbent assay; HAQ: health assessment questionnaire; HLA: human leukocyte antigen; HR: hazard ratios; Ig: immunoglobulin; JIA: juvenile idiopathic arthritis; MI: myocardial infarction; PROSPER: PROspective Study of Pravastatin in the Elderly at Risk; RA: rheumatoid arthritis; RF: rheumatoid factor; SD: standard deviation; SNP: single nucleotide protocol; TIA: transient ischaemic attack.

## Competing interests

The authors declare that they have no competing interests.

## Authors' contributions

RM, FK, ZdJ, JvN and ST made the acquisition of data. JvN, RM, ST and AvdH carried out the analysis and interpretation of data. JvN, RM, ST and AvdH have been involved in drafting the manuscript. AvdH, TH, WJ and RT were responsible for revising critically the manuscript for important intellectual content. All authors have given final approval of the version to be published.
